# A Proof-of-Concept Clinical Trial of A Single Luteal Use of Long-Acting Gonadotropin-Releasing Hormone Antagonist Degarelix in Controlled Ovarian Stimulation for *In Vitro* Fertilization: Long Antagonist Protocol

**DOI:** 10.3389/fendo.2018.00025

**Published:** 2018-03-01

**Authors:** Evangelos G. Papanikolaou, Hakan Yarali, Evi Timotheou, Michael Grynberg, Odysseas Zafeiratis, Herman Tournaye, Robert Najdecki

**Affiliations:** ^1^Centre of Reproduction and Genetics, Assisting Nature, Thessaloniki, Greece; ^2^3rd Department of Obstetrics and Gynecology, Aristotle University of Thessaloniki, Thessaloniki, Greece; ^3^Hacetepe University of Ankara, Ankara, Turkey; ^4^Hôpital Jean-Verdier, Université Paris-Sud, Bondy, France; ^5^Free University of Brussels (VUB), Brussels, Belgium

**Keywords:** in *vitro* fertilization, antagonist protocol, degarelix, ovarian stimulation, long agonist protocol, pregnancy, long antagonist protocol

## Abstract

**Introduction:**

A drawback of gonadotropin-releasing hormone (GnRH) antagonist protocols in *in vitro* fertilization (IVF) is that they have limited flexibility in cycle programming. This proof of concept study explored the efficacy of a single-dose, long-acting GnRH antagonist IVF protocol. Trial registration number is NCT03240159, retrospectively registered on March 08, 2017.

**Materials and methods:**

The efficacy of a single-dose long-acting antagonist, degarelix, was explored initially in healthy donors and subsequently in infertile patients. In the first part, five healthy oocyte donors underwent ovarian stimulation with this new protocol: in the late luteal phase, at day 24, a bolus injection of degarelix was administered subcutaneously to control the LH surge in the follicular phase. Ovarian stimulation with gonadotropins was initiated subsequently from day 7 to day 10. End points were first to inhibit the LH surge later in the follicular phase and, second, to retrieve mature oocytes for IVF. In the second part, five infertile women received the same bolus injection of degarelix administered during the luteal phase at day 24. Different gonadotropin starting days (day 2 through day 8) were tested in order to observe possible differences in ovarian stimulation. In these infertile patients, fresh embryo transfers were performed to assess the pregnancy efficacy of this protocol on pregnancy outcomes and to address any possible negative effects on endometrium receptivity.

**Results:**

In the first part of the study, all donors were effectively downregulated with a single luteal dose of 0.5 ml of degarelix for up to 22 days until the final oocyte maturation triggering day. Mature oocytes were retrieved after 36 h from all patients and all produced 2–7 blastocysts. In the second part, all five infertile patients achieved sufficient LH downregulation and completed ovarian stimulation without any LH surge. All patients (except one with freeze all strategy) had blastocysts transferred and pregnancy occurred in three out of five women.

**Conclusion:**

A single dose of the long-acting antagonist degarelix during the luteal phase appears to be effective in downregulating hypophysis during ovarian stimulation. This represents a possible new protocol for IVF, which should be further elucidated in RCTs.

## Introduction

In contrast to gonadotropin-releasing hormone (GnRH) agonist treatment, GnRH antagonists block GnRH receptors by competitive binding, resulting in immediate gonadotrophin suppression. This enables a short treatment regimen for ovarian stimulation as well as immediate recovery from hypophysis within hours after the antagonist injection. Disadvantages with the use of GnRH agonists, such as ovarian cyst formation, vaginal spotting, hot flushes, headaches, tiredness, and dizziness, are completely eliminated when a GnRH antagonist is used (1, 2). Furthermore, there is solid evidence that the use of a GnRH antagonist is associated with a substantial reduction in ovarian hyperstimulation syndrome (OHSS) risk compared with long-course GnRH agonist protocols (3), without reducing the likelihood of achieving live birth (4).

Despite the clear benefits associated with GnRH antagonist protocols (resulting in a steady increase in their use), GnRH agonists are still the treatment of choice in the majority of assisted reproductive technology clinics (5, survey 2010). It seems that the major reason for favoring a GnRH agonist protocol is primarily that GnRH antagonists offer less flexibility in cycle programming. A secondary factor is the asynchrony of the follicular cohort compared with the long GnRH agonist protocol (6).

Programming of antagonist cycles continues to be a challenge. The use of oral contraceptive pill pretreatment to achieve a better synchronized response and a more scheduled cycle unfortunately has been associated with significantly lower ongoing pregnancy rates, longer durations of stimulation, and higher gonadotropin consumption (7). A pretreatment with GnRH antagonists during three consecutive days, or a varying number of days (i.e., 2, 3, or 4 days), in accordance with the intended onset of ovarian stimulation, has been proposed in a pilot study as a convenient tool to schedule GnRH antagonist cycles (8), but it has not been tested in larger studies. Moreover, with current GnRH antagonist protocols daily administration of antagonists is determined due to dose and half-life.

Recently, a long-acting GnRH antagonist, degarelix (Ferring Pharmaceuticals, Germany), was introduced for the treatment of prostatic cancer (9, 10). We hypothesized that in order to improve the flexibility of GnRH antagonist protocols, the administration of a single-dose, long-acting antagonist in the late luteal phase of the preceding cycle (at day 24) could effectively downregulate endogenous gonadotropins and synchronize antral follicles, and thus, allowing delayed initiation of ovarian stimulation and euploid embryos production. We therefore investigated the efficacy of degarelix in two populations (healthy oocyte donors and infertile patients). Initially, healthy donors were recruited with the objective being the sufficient suppression of LH with concurrent production of mature oocytes and implantable blastocysts. In a second part of the study, efficacy on pregnancy outcomes in infertile patients was explored using different gonadotropin starting days. Implantation potential was also tested with synchronous fresh embryo transfers.

## Materials and Methods

The current study was a proof of concept study, which was executed in two parts. Ten female patients were recruited: five oocyte donors and five infertile patients. They all signed an informed consent waiver and underwent all the proper evaluation in order to be recruited. An Institutional Review Board approval was given to the protocol. Trial registration number is NCT03240159 and is retrospectively registered in order to protect the patent of the protocol, as Papanikolaou protocol. The study was carried out at a private *in vitro* fertilization (IVF) clinic: Assisting Nature, Center of Assisted Reproduction & Genetics, Thessaloniki, Greece.

In first part of the study, we applied this protocol to five (*n* = 5) young healthy women, undergoing IVF for oocyte donation. They had previously undergone ovarian stimulation with antagonist protocol and therefore were familiar with the IVF procedure. Inclusion criteria for donors were as follows: an age of <36 years, an AMH level of >2 ng/ml, proven fertility, absence of polycystic ovaries, and without an exclusionary medical history. In the second part of the study, five (*n* = 5) infertile women were recruited. Inclusion criteria were as follows: (a) an age of <39 years, (b) a mean cycle length of 28–30 days, (c) availability of fresh ejaculated partner sperm, (d) absence of polycystic ovaries, (e) normal follicle-stimulating hormone (FSH) and TSH, (f) and a normal uterine cavity as assessed by echography (Table 1).

**Table 1:** Patients enrolled in the Long Antagonist Protocol proof of concept study.

	Age	AMH (ng/ml)	Luteal day antagonist	Menses	FSH day initiation	Stimulation days	Triggering	COCs	MII	2PN	Total embryos	Pregnancy outcome
**A study OD**						**OD patients**						

ID-01	28	2.5	d-24	d-27	CycDay10	11	GnRH	10	8	6	2 D5	Heterotopic
ID-02	24	4.59	d-26	d-28	CycDay08	10	GnRH	29	22	16	10 D5	Ongoing
ID-03	28	2.1	d-24	d-27	CycDay06	10	GnRH	15	15	14	6 D5	Ongoing
ID-04	31	2.8	d-24	d-26	CycDay09	11	GnRH	17	10	10	4 D5	Negative
ID-05	26	2.0	d-24	d-26	CycDay07	11	GnRH	13	11	10	2 D5	Ongoing

**B study IVF**						**IVF patients**						

ID-06	30	3.49	d-24	d-26	CycDay08	10	rec-HCG	16	12	11	6 D5	Delivery
ID-07	34	3.0	d-24	d-28	CycDay06	10	rec-HCG	16	13	9	4 D5	Clinical
ID-08	39	2.4	d-24	d-27	CycDay04	11	rec-HCG	6	6	6	3 D5	Negative
ID-09	27	4.48	d-26	d-28	CycDay03	11	GnRH	22	19	19	16 D5	Frall/Clinical
ID-10	33	1.47	d-24	d-26	CycDay02	11	rec-HCG	7	5	3	2 D5	Clinical

*FSH, follicle-stimulating hormone; GnRH, gonadotropin-releasing hormone; HCG, human chorionic gonadotropin; IVF, in vitro fertilization*.

All patients on day 24 of the preceding cycle, upon confirmation of negative b-human chorionic gonadotropin (HCG) blood levels, received a single 0.5 ml (10 mg) subcutaneous injection of the new long-acting antagonist, degarelix (Firmagon^®^, Ferring)—one patient received 0.6 ml. Each degarelix vial contains 80 mg of powder and 4.2 ml of water for injection. Vials were kept in an upright position and gently swirled until the liquid looks clear and without undissolved powder. After reconstitution, 4 ml of 80 mg degarelix was achieved. The reconstitution needle was then exchanged for an administration needle (27 G/1-1/4 in) for deep subcutaneous injection. Any air bubbles were also removed. The injection was given by a midwife at our clinic.

**Figure 1:**
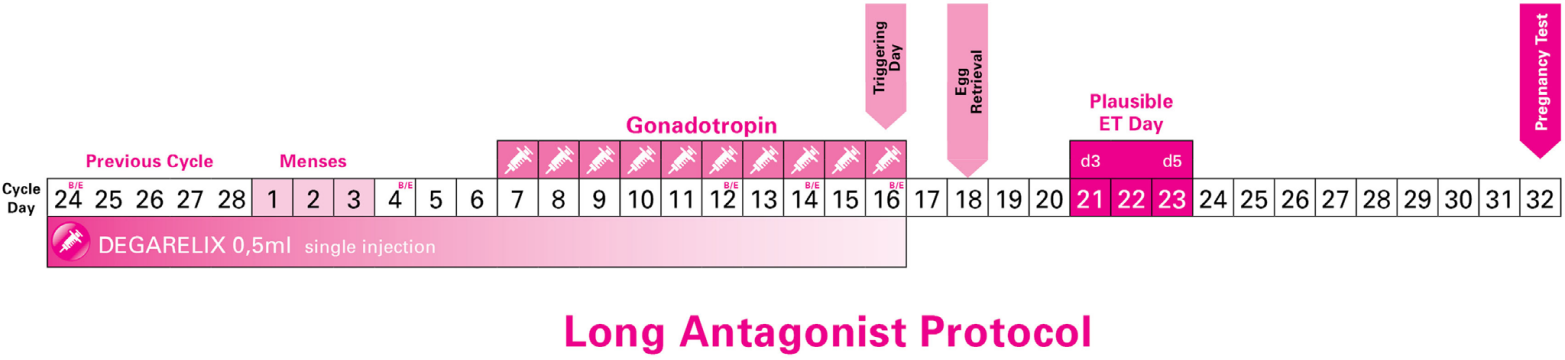
Long Antagonist Protocol with flexible starting day of gonadotropin (just indicative in the current image).

After 2–5 days, all patients had their menses. On day 2 up to day 4 of their new cycle, all patients were assessed (echography and hormone levels) to confirm downregulation of LH and absence of any ovarian cysts (Figure 1).

Each study participant, including both donors and infertile patients, was instructed to initiate ovarian stimulation from the cycle day 2 (CD2) up to and including the cycle day 10 (CD10): CD10 (*n* = 1), CD9 (*n* = 1), CD8 (*n* = 2), CD7 (*n* = 1), CD6 (*n* = 2), CD4 (*n* = 1), CD3 (*n* = 1), and CD2 (*n* = 1). This was decided in order to test the duration and flexibility of this new protocol without risking an LH surge. For this reason, LH, E2, and P4 levels were strictly monitored. All hormone tests were performed using a Roche 6000 series Cobas analyzer. Detection rates were as follows: 5–3,000 pg/ml for estradiol, 0.1–200 mIU/ml for LH, and 0.05–60 ng/ml for progesterone.

Ovarian stimulation was initiated with either human menopausal gonadotropins or recombinant FSH at a dose of 225–300 IU daily. This first gonadotropin injection day was considered as stimulation day 1 (SD1). After 5 days, on SD6 all patients underwent ultrasound examination and hormone level testing. Then within 2 or 3 days a third evaluation was done. After 1 or 2 days a fourth evaluation was performed until three follicles had reached 18 mm in size. No other GnRH antagonist injection was permitted.

Final oocyte maturation was triggered with either recombinant HCG 250 mcg (IVF patients) or 0.3 mg of triptorelin (OD patients), and after 36 h oocyte retrieval took place. In all patients, fresh ejaculated sperm was used, and intracytoplasmic sperm injection was performed 40 h after triggering injection. All embryos were cultured in sequential media (fertilization medium, cleavage medium, and blastocyst medium) provided by Origio. All embryos were incubated in incubator G210 by K-Systems. Only blastocysts were transferred or cryopreserved. Blastocyst cryopreservation was performed using the Kitazato vitrification kit.

A positive b-HCG blood test (above 10 mIU/ml) was used to confirm pregnancy. Clinical pregnancy was defined as a positive heart beat at 7 weeks, and ongoing pregnancy was defined as positive heart beat at 12 weeks.

In the first part study (oocyte donors), the primary end point was the incidence of an LH surge in the follicular phase. Secondary end points were as follows: the retrieval of mature oocytes, fertilization rate, formation of blastocysts, and protocol tolerability. In the second part of the study, the primary end points were as follows: (a) incidence of LH surge and (b) occurrence of pregnancy. Secondary end points were as follows: incidence of an LH surge in the follicular phase, blastulation rate, and tolerability of the protocol for patients.

## Results

In both phases of the study, all patients received a bolus injection of degarelix. Additional antagonist doses later in the follicular phase during multifollicular ovarian stimulation were not necessary.

**Figure 2:**
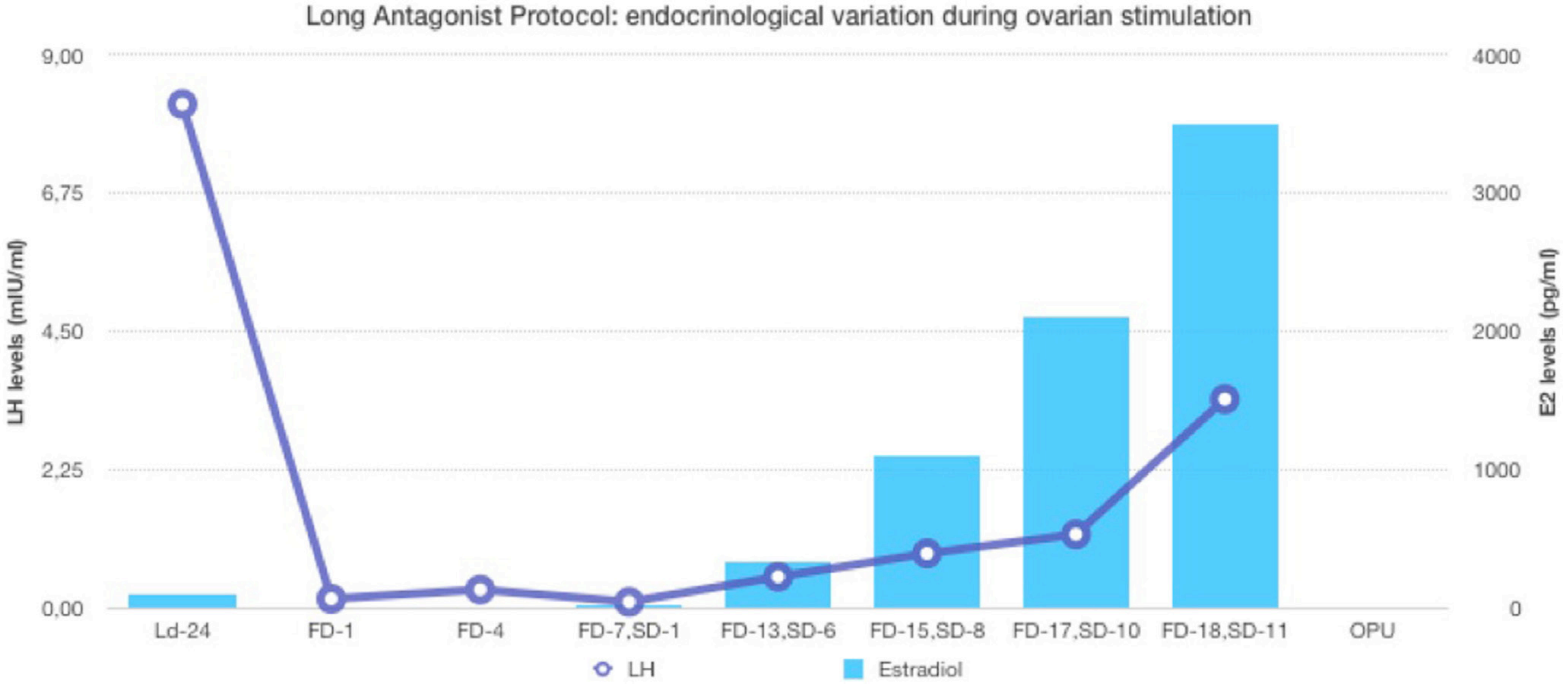
Endocrinological data (E2 and LH variation) during long antagonist protocol (patient ID-05).

In the first part of the study, the age of the five donors ranged from 24 to 31 years, and all BMI scores were below 29. No LH surge was noticed throughout the cycle, and no additional antagonist was needed throughout the period of downregulation in all donors. The maximum length of the follicular phase including ovarian stimulation without an LH surge reached 22 days. In all patients mature oocytes were found; the fertilization rate was 75%, and the mean number of blastocysts produced was 4.8 (Table 1). Five synchronized oocyte acceptors received two blastocysts per transfer and four became pregnant. Unfortunately, one patient had a heterotopic pregnancy and needed to undergo laparoscopy for the extrauterine sac (as the endometrial pregnancy had ended in miscarriage). In that particular patient, her partner’s sperm quality was extremely poor, 1–2 motile spermatozoa per field. Sixty percentage of the acceptors (three out of five) had ongoing pregnancy. Endocrinological data (LH, E2, and Prog) for each patient are summarized in Table 2. Downregulation of hypophysis *via* the single luteal bolus of degarelix is shown in Figure 2.

**Table 2:** Stimulation days, hormone tests, degarelix injection day, and gonadotropin starting day.

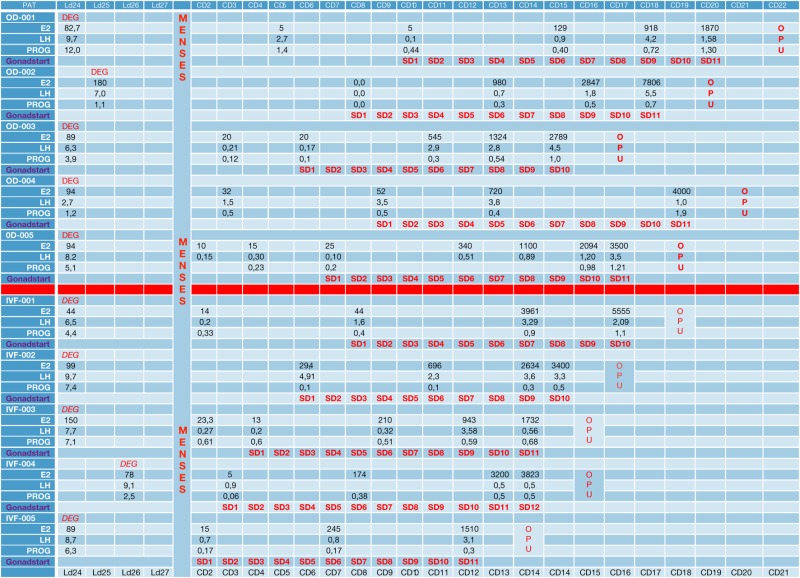

*CD, cycle day; SD, stimulation day*.

Infertile patients recruited for the second part of the study were aged 27–39 years, and their BMI was between 24 and 31. Three patients had male factor infertility, one had unexplained infertility, and one had tubal infertility. All IVF patients were similarly and effectively downregulated, and the flexibility with the protocol was excellent, allowing for initiation of gonadotropin from the CD2 up to the CD8. All patients produced cumulus oocyte complexes (range: 6–22); all patients produced blastocysts (range: 3–16); and all except one underwent fresh embryo transfer (Table 3). One woman did not have embryo transfer in order to avoid OHSS, and all her embryos were frozen. She became pregnant in the subsequent frozen cycle. In three women out of four undergoing transfer a pregnancy occurred, and all three pregnancies were ongoing (60% per protocol analysis and 80% per patient). Endocrinological data for each IVF patient are summarized in Table 2, including cycle day initiation, duration of stimulation (10–11 days), gonadotropin dosing, follicular development, and triggering of final oocyte maturation.

**Table 3:** Embryological parameters and pregnancy outcome.

	OD patients (*n* = 5)	*In vitro* fertilization patients (*n* = 5)
Mean age (years ± SD)	27.4 ± 5.7	32.6 + 6.3
Stimulation days (range)	10–11	10–12
MII oocytes (median)	14	11
Fertilization rate	75%	78%
Total blastocysts produced (mean ± SD)	4.8 ± 1.1	7 ± 2.3
LH surge	None	None
Pregnancy outcome (fresh ET)	4/5	3/5
Cumulative pregnancy outcome	3/5	4/5

In terms of tolerability of the protocol, all women (both donors and IVF patients) complained about site edema and itching. One IVF patient complained of stomach pain and vomiting, although these occurred several days after the injection.

## Discussion

Despite the patient-friendly profile of an antagonist protocol with fewer injections, fewer days of stimulation and a lower risk of OHSS, doctors have still not adopted this treatment protocol. The main reason is that an antagonist protocol is not considered to be doctor friendly due to its flexibility constraints, for example, the difficulty in scheduling oocyte retrievals and avoiding weekends.

The use of a long-acting GnRH antagonist protocol seems to address the flexibility issue with the antagonist protocols. A single injection promotes thorough suppression of LH and maintains low levels of estradiol (Table 2). Consequently, low endogenous LH and FSH levels with a single antagonist dose allow iatrogenic follicular stimulation with exogenously administered FSH and/or LH, with minimal risk for premature LH surges and premature ovulation, as our results have confirmed (Table 2; Figure 2). None of our patients ovulated or had premature LH surges up to 24 days after the initial administration of the antagonist in the luteal phase, which was the ideal outcome.

Our current proof of concept study shows that the potential disadvantages of a GnRH antagonist protocol over GnRH agonist protocols, in terms of the less flexible options for cycle programming, are eliminated by single use of long-acting GnRH antagonists during the luteal phase. All 10 patients who were enrolled in both donor and infertile groups started follicular stimulation from CD3 up to CD10.

Degarelix is only for subcutaneous use (11). It is available in either 80 or 120 mg doses. We used the 80 mg vial. After reconstitution, each vial contains 4 ml where each milliliter contains 20 mg, with each 0.1 ml containing 2 mg. Degarelix was administered with an insulin syringe. We selected the degarelix dose of 0.5 ml or 10 mg empirically based on preliminary testing and published clearance studies in primates and in human males (10). Only one preliminary study has been performed in human females (12). In males treated for prostate cancer, side effects including hot flushes and injection site pain and swelling were present.

Our approach is similar in concept to the long GnRH agonist protocol and yet maintains the advantages of the GnRH antagonist protocol, which is mainly the decreased risk of OHSS. Moreover, the risk for OHSS can be almost eliminated by using a GnRH agonist for triggering. We believe that our novel protocol could optimize follicular recruitment, as 91% of the oocytes were mature. In addition, the LH level dropped significantly and was maintained below 14 mIU/ml throughout without needing any additional GnRH antagonist dosing (Table 3).

However, many parameters for this new protocol still need to be established. First, which day during the luteal phase is ideal for administration (the later the day, the lower the risk of LH surges in the late follicular phase)? An earlier date, toward luteal day 21 may enable better follicular recruitment. Second, which dose should be administered? The dose should not be too high as this could oversuppress endogenous LH and FSH, which would result in longer stimulation and greater FSH consumption. Third, on which day should FSH administration during the follicular phase be initiated? There is a trend in our study, although preliminary, that the earlier gonadotropin is started, the longer the simulation, reaching up to 11 days. All these aspects will need to be established in large, well-designed studies to optimize this new protocol.

We performed this trial as a proof of concept in order to establish the efficacy of a new protocol, which we believe will revolutionize IVF practice in the future. The 60% pregnancy rate in both parts of the study is a good indicator of efficacy. Nevertheless, the limitations of this study are that it had a small population and had no comparative control group, preventing extrapolation of the findings to a larger scale IVF population. Moreover, the cost of degarelix is relatively higher; in Greece one vial of 80 mg degarelix costs 100 euros whereas five vials of ganirelix or cetrorelix cost 50 euros approximately. This might be sorted out in the future by the manufacturing company if they develop lower dose vials only for use in IVF as it happened with GnRH agonists years ago for endometriosis, IVF, and prostate cancer.

This novel approach appears to offer a promising alternative to the long-established GnRH agonist regimens for all IVF couples. Moreover, this new long antagonist protocol could be used in oocyte donors to facilitate synchronization with the acceptor without risking OHSS. Additionally, a single dose of antagonist can facilitate the hormone replacement protocol for frozen embryos, which is becoming increasingly popular, particularly after the freeze-all strategy. However, further studies are required to confirm this novel concept prior to its universal implementation in IVF practice.

## Conclusion

This proof of concept study indicates that using a single luteal phase antagonist protocol could potentially combine the benefits of both antagonist and agonist protocols. For example, this approach could combine the flexibility of programming and homogenized follicular cohort benefits of the long agonist protocol with the near eradication of OHSS and the patient friendliness of the short antagonist protocol.

## Ethics Statement

This study was carried out in accordance with the recommendations of “name of guidelines, name of committee” with written informed consent from all subjects. All subjects gave written informed consent in accordance with the Declaration of Helsinki. The protocol was approved by the Institutional Review Board of Assisting Nature, Centre of Reproduction and Genetics.

## Author Contributions

All authors performed the study, analysis, edited the manuscript.

## Conflict of Interest Statement

The authors declare that the research was conducted in the absence of any commercial or financial relationships that could be construed as a potential conflict of interest. The reviewer AC and handling editor declared their shared affiliation.

## Abbreviations

ART, assisted reproductive technology; FSH, follicle-stimulating hormone; IVF, *in vitro* fertilization; ICSI, intracytoplasmic sperm injection; GnRH, gonadotropin-releasing hormone; HCG, human chorionic gonadotropin; OHSS, ovarian hyperstimulation syndrome.
